# COVID-19 Surveillance After Expiration of the Public Health Emergency Declaration ― United States, May 11, 2023

**DOI:** 10.15585/mmwr.mm7219e1

**Published:** 2023-05-12

**Authors:** Benjamin J. Silk, Heather M. Scobie, William M. Duck, Tess Palmer, Farida B. Ahmad, Alison M. Binder, Jodi A. Cisewski, Seth Kroop, Karl Soetebier, Meeyoung Park, Aaron Kite-Powell, Andrea Cool, Erin Connelly, Stephanie Dietz, Amy E. Kirby, Kathleen Hartnett, Jocelyn Johnston, Diba Khan, Shannon Stokley, Clinton R. Paden, Michael Sheppard, Paul Sutton, Hilda Razzaghi, Robert N. Anderson, Natalie Thornburg, Sarah Meyer, Caryn Womack, Aliki P. Weakland, Meredith McMorrow, Lanson R. Broeker, Amber Winn, Aron J. Hall, Brendan Jackson, Barbara E. Mahon, Matthew D. Ritchey

**Affiliations:** ^1^Coronavirus and Other Respiratory Viruses Division, National Center for Immunization and Respiratory Diseases, CDC; ^2^Office of Public Health Data, Surveillance, and Technology, CDC; ^3^Office of Innovation and Analytics, Agency for Toxic Substances and Disease Registry, Atlanta, Georgia; ^4^Division of Vital Statistics, National Center for Health Statistics, CDC; ^5^Division of Healthcare Quality and Promotion, National Center for Emerging and Zoonotic Diseases, CDC; ^6^Division of Emergency Operations, Center for Preparedness and Response, CDC; ^7^Booz Allen Hamilton, McLean, Virginia; ^8^Division of Foodborne, Waterborne, and Environmental Diseases, National Center for Emerging and Zoonotic Diseases, CDC; ^9^Immunization Services Division, National Center for Immunization and Respiratory Diseases, CDC.

On January 31, 2020, the U.S. Department of Health and Human Services (HHS) declared, under Section 319 of the Public Health Service Act, a U.S. public health emergency because of the emergence of a novel virus, SARS-CoV-2.* After 13 renewals, the public health emergency will expire on May 11, 2023. Authorizations to collect certain public health data will expire on that date as well. Monitoring the impact of COVID-19 and the effectiveness of prevention and control strategies remains a public health priority, and a number of surveillance indicators have been identified to facilitate ongoing monitoring. After expiration of the public health emergency, COVID-19–associated hospital admission levels will be the primary indicator of COVID-19 trends to help guide community and personal decisions related to risk and prevention behaviors; the percentage of COVID-19–associated deaths among all reported deaths, based on provisional death certificate data, will be the primary indicator used to monitor COVID-19 mortality. Emergency department (ED) visits with a COVID-19 diagnosis and the percentage of positive SARS-CoV-2 test results, derived from an established sentinel network, will help detect early changes in trends. National genomic surveillance will continue to be used to estimate SARS-CoV-2 variant proportions; wastewater surveillance and traveler-based genomic surveillance will also continue to be used to monitor SARS-CoV-2 variants. Disease severity and hospitalization-related outcomes are monitored via sentinel surveillance and large health care databases. Monitoring of COVID-19 vaccination coverage, vaccine effectiveness (VE), and vaccine safety will also continue. Integrated strategies for surveillance of COVID-19 and other respiratory viruses can further guide prevention efforts. COVID-19–associated hospitalizations and deaths are largely preventable through receipt of updated vaccines and timely administration of therapeutics (*1**–**4*).

Although COVID-19 no longer poses the societal emergency that it did when it first emerged in late 2019, COVID-19 remains an ongoing public health challenge. By April 26, 2023, more than 104 million U.S. COVID-19 cases, 6 million related hospitalizations, and 1.1 million COVID-19–associated deaths were reported to CDC and summarized on CDC’s COVID Data Tracker.^†^ COVID-19 was the third leading cause of death during 2020 and 2021^§^ and the fourth leading cause during 2022 (*5*). To mitigate the consequences of the pandemic, approximately 675 million COVID-19 vaccine doses were administered, including 55 million updated (bivalent) booster doses. Based on seroprevalence data, infection- and vaccine-induced population immunity in the United States was 95% by December 2021 (*6*). As a result, rates of COVID-19–associated hospitalizations and deaths have declined substantially since March 2022 (*7*). This report describes changes to the national COVID-19 surveillance strategy, data sources, and indicators that will be made after the public health emergency declaration expires; these indicators will be displayed as weekly or otherwise scheduled updates to CDC’s COVID Data Tracker.

## Continued Surveillance After May 11, 2023

Most COVID-19 surveillance data sources will continue to be available after the public health emergency declaration ends on May 11; the reporting cadence of some will change, and three will be discontinued (Table 1). Since December 15, 2022, daily reporting to CDC’s National Healthcare Safety Network (NHSN) of aggregate counts of patients with laboratory-confirmed COVID-19 admitted to acute care and critical access U.S. hospitals has been required. After the public health emergency ends on May 11, 2023, a switch to a weekly cadence of national reporting will affect data processing and introduce reporting lag. NHSN data on COVID-19 hospital admissions per 100,000 population will be the primary surveillance indicator to help guide community and individual decisions related to risk and prevention behaviors. These data have similar suitability for tracking local COVID-19 activity as do COVID-19 Community Levels (CCLs) (*8*) and will be updated weekly on COVID Data Tracker at the county,^¶^ state, regional, and national levels.

**TABLE 1:** Changes to national reporting of COVID-19 surveillance data sources and accessibility after the expiration of the public health emergency declaration — United States, May 11, 2023

Surveillance data source	Changes to COVID Data Tracker	Accessibility*
**National Healthcare Safety Network^†^**	Continued availability with weekly updates	COVID Data Tracker
**National Vital Statistics System^§^**	New availability with weekly updates	COVID Data Tracker
**National Syndromic Surveillance Program^¶^**	Continued availability with weekly updates	COVID Data Tracker
National Respiratory and Enteric Viruses Surveillance System**	New availability with weekly updates	COVID Data Tracker
**National SARS-CoV-2 Strain Surveillance^††^**	Continued availability with biweekly updates	COVID Data Tracker
**National Wastewater Surveillance System^§§^**	Continued availability with daily or weekly updates	COVID Data Tracker
**Traveler-based Genomic Surveillance program^¶¶^**	Continued availability with weekly updates	COVID Data Tracker
COVID-NET (sentinel surveillance for COVID-19–associated hospitalizations)***	Continued availability with weekly updates	COVID Data Tracker
**Large health care databases^†††^**	Continued availability with monthly or quarterly updates	COVID Data Tracker
**COVID-19 vaccination administration^§§§^**	Continued availability with monthly updates	COVID Data Tracker
**National Immunization Survey^¶¶¶^**	Continued availability with weekly or monthly updates	COVID Data Tracker and COVIDVaxView
Line-level COVID-19 case reporting****	Continued availability with weekly updates	COVID Data Tracker and https://data.cdc.gov
**CELR^††††^**	Discontinued on May 11, 2023	Archived on https://healthdata.gov
**ACDC reporting^§§§§^**	Discontinued on May 11, 2023	Archived on https://data.cdc.gov
**Community transmission level and COVID-19 community level metrics**	Discontinued on May 11, 2023	Archived on https://data.cdc.gov

**Abbreviations:** ACDC = aggregate cases and death counts; CELR = COVID-19 electronic laboratory reporting; COVID-NET = Coronavirus Disease 2019-Associated Hospitalization Surveillance Network.

* https://covid.cdc.gov/covid-data-tracker; https://www.cdc.gov/vaccines/imz-managers/coverage/covidvaxview/index.html; https://data.cdc.gov;https://healthdata.gov

^†^ https://www.hhs.gov/sites/default/files/covid-19-faqs-hospitals-hospital-laboratory-acute-care-facility-data-reporting.pdf

^§^ https://www.cdc.gov/nchs/nvss/index.htm

^¶^ https://www.cdc.gov/nssp/index.html

** https://www.cdc.gov/surveillance/nrevss/index.html

^††^ https://covid.cdc.gov/covid-data-tracker/#variant-proportions

^§§^ https://www.cdc.gov/nwss/index.html

^¶¶^ https://wwwnc.cdc.gov/travel/page/travel-genomic-surveillance

*** https://www.cdc.gov/coronavirus/2019-ncov/covid-data/covid-net/purpose-methods.html

^†††^ https://covid.cdc.gov/covid-data-tracker/index.html#hospitalizations-severity

^§§§ ^https://www.cdc.gov/vaccines/covid-19/reporting/index.html; https://www.cdc.gov/coronavirus/2019-ncov/vaccines/reporting-vaccinations.html

^¶¶¶^ https://www.cdc.gov/vaccines/imz-managers/nis/index.html

**** https://cdn.ymaws.com/www.cste.org/resource/resmgr/ps/ps2022/22-ID-01_COVID19.pdf

^††††^ https://www.cdc.gov/coronavirus/2019-ncov/lab/reporting-lab-data.html

^§§§§^ ACDC data are compiled using automated data extraction from state and jurisdictional websites and dashboards and direct submissions from jurisdictions. ACDC data shifted from daily to weekly cadence in October 2022, with some jurisdictions continuing to report daily totals and others reporting only weekly totals.

Considerable gains in the timeliness of National Vital Statistics System (NVSS) death certificate data processing were accomplished during the pandemic (*9*). Provisional death certificate data from NVSS, including decedents for whom COVID-19 is listed as an underlying or a contributing cause of death, will be the primary data source for monitoring COVID-19 mortality. Among several mortality-based metrics, the percentage of COVID-19–associated deaths among all reported deaths in NVSS will be a new weekly surveillance indicator on COVID Data Tracker that is comparable with a corresponding influenza mortality surveillance indicator (Table 2).** Because the lag in mortality reporting is similar for COVID-19 deaths and deaths overall, this indicator is not biased by incomplete reporting in previous weeks and allows for timely tracking of mortality trends (*8*).

**TABLE 2:** Data sources, metrics, and geographic levels for continued COVID-19 surveillance after the expiration of the public health emergency declaration — United States, May 11, 2023

**Surveillance data source**	**Metrics**	**Geographic level**
National Healthcare Safety Network*	Count of COVID-19–associated hospital admissions	County, state, regional per HHS regions, and national
	Rate of COVID-19–associated hospital admissions per 100,000 population	
	% Change in COVID-19–associated hospital admissions from previous wk	
	% of inpatient beds occupied by COVID-19 patients	
	Absolute change in % of inpatient beds occupied by COVID-19 patients from previous wk	
	% of ICU beds occupied by COVID-19 patients	
	Absolute change in % of ICU beds occupied by COVID-19 inpatients from previous wk	
**National Vital Statistics System^†^**	% of all COVID-19–associated deaths	State, regional per HHS regions, and national
	Count of COVID-19–associated deaths	
	Rate of COVID-19–associated deaths per 100,000 population (crude and age-adjusted)	
**National Syndromic Surveillance Program^§^**	% of ED visits with COVID-19 discharge diagnoses	Select states, regional per HHS regions, and national
	% Change in % of ED visits with COVID-19 from previous wk	
**National Respiratory and Enteric Viruses Surveillance System^¶^**	SARS-CoV-2 NAAT % positivity	Regional per HHS regions and national
	% Change in SARS-CoV-2 NAAT % positivity from previous wk	
National SARS-CoV-2 Strain Surveillance**	Weighted and nowcast estimates of variant proportions	Regional per HHS regions and national
**National Wastewater Surveillance System^††^**	Current virus levels in wastewater by site	Select counties, states, regional per HHS regions, and national
	% Change in the last 15 days	
	% of wastewater samples with detectable virus	
	Predominant variants by site	
	Relative abundance of variants by site	
**Traveler-based Genomic Surveillance program^§§^**	% Test positivity (pooled)	National
	Variant proportions among positive pools	
**COVID-NET (sentinel surveillance for COVID-19–associated hospitalizations)^¶¶^**	Rate of COVID-19–associated hospital admissions	Select states and counties (sentinel network)
	COVID-19–associated hospital admissions rates, by age group, sex, and race and ethnicity	
	% of COVID-19–associated hospitalizations by underlying medical conditions	
	% of COVID-19–associated hospitalizations resulting in ICU, IMV, or death	
Large health care databases***	% of hospitalized COVID-19 patients who were admitted to an ICU	Select participating health care organizations
	% of hospitalized COVID-19 patients who received IMV	
	% of hospitalized COVID-19 patients who died	
**COVID-19 vaccinations^†††^**	% Up-to-date status	57 of 64 jurisdictions (as of April 25, 2023)
**National Immunization Survey^§§§^**	% Vaccinated and intent for vaccination	National (adults and children); state (adults)
	Vaccination status and intent by demographic characteristics and behavioral indicators	

**Abbreviations:** COVID-NET = Coronavirus Disease 2019-Associated Hospitalization Surveillance Network; ED = emergency department; HHS = U.S. Department of Health and Human Services; ICU = intensive care unit; IMV = invasive mechanical ventilation; NAAT = nucleic acid amplification test.

* https://www.hhs.gov/sites/default/files/covid-19-faqs-hospitals-hospital-laboratory-acute-care-facility-data-reporting.pdf.

^†^ https://www.cdc.gov/nchs/nvss/index.htm

^§^ https://www.cdc.gov/nssp/index.html

^¶^ https://www.cdc.gov/surveillance/nrevss/index.html

** https://covid.cdc.gov/covid-data-tracker/#variant-proportions

^††^ https://www.cdc.gov/nwss/index.html

^§§^ https://wwwnc.cdc.gov/travel/page/travel-genomic-surveillance.

^¶¶^ https://www.cdc.gov/coronavirus/2019-ncov/covid-data/covid-net/purpose-methods.html

*** https://covid.cdc.gov/covid-data-tracker/index.html#hospitalizations-severity

^†††^ https://www.cdc.gov/vaccines/covid-19/reporting/index.html; https://www.cdc.gov/coronavirus/2019-ncov/vaccines/reporting-vaccinations.html; https://www.cdc.gov/coronavirus/2019-ncov/vaccines/stay-up-to-date.html

^§§§^ https://www.cdc.gov/vaccines/imz-managers/nis/index.html

The National Syndromic Surveillance Program (NSSP) has expanded substantially during the COVID-19 pandemic, with data from 6,300 facilities in all 50 states, the District of Columbia, and Guam. NSSP includes 75% of all U.S. ED visits (Table 1); coverage is currently limited in Minnesota and Oklahoma, and discharge diagnosis completeness is currently limited in Missouri. Using NSSP discharge diagnosis data, the weekly percentage of patients who receive a diagnosis of COVID-19 among all ED visits is an indicator that can identify trends earlier than hospital admission rates can (*8*).

Monitoring national and regional trends in the percentage of positive SARS-CoV-2 nucleic acid amplification test (NAAT) results will be based on surveillance data from the National Respiratory and Enteric Virus Surveillance System (NREVSS). This system is an established sentinel network of approximately 450 clinical, public health, and commercial laboratories that voluntarily submit weekly data on numbers of positive test results and total tests performed. As another early indicator, the percentage of positive SARS-CoV-2 test results from NREVSS is a suitable alternative to that obtained through COVID-19 electronic laboratory reporting (CELR), which will not be possible after May 11, because reporting of negative rest results will not be required (*8*). Because SARS-CoV-2 testing volumes and the geographic representation of NREVSS laboratories are heterogeneous (with one to 31 participating laboratories per state), region-level rather than state-level data will be displayed.

Genomic surveillance to estimate SARS-CoV-2 variant proportions at the national and regional levels will continue with a biweekly cadence and revised analytic methods for weighting based on the probability of selecting positive laboratory specimens for sequencing (*10*). As fewer specimens and sequences become available, a system that is scaled sufficiently to allow for regional estimates will be established in collaboration with the network of public health laboratories that have participated in the National SARS-CoV-2 Strain Surveillance program.^††^ The National Wastewater Surveillance System (NWSS) and Traveler-based Genomic Surveillance (TGS) Program are additional sources of surveillance data for monitoring early trends in SARS-CoV-2 infections and variant proportions (NWSS) and for early detection of new variants in travelers entering the United States (TGS) that will continue to be available with daily or weekly updates.^§§^

In addition to NHSN, sentinel surveillance and large health care databases will continue to monitor disease severity and hospitalization-related outcomes. The Coronavirus Disease 2019–Associated Hospitalization Surveillance Network (COVID-NET)^¶¶^ uses active, population-based surveillance to estimate rates of laboratory-confirmed COVID-19–associated hospital admissions and also collects detailed clinical information, including underlying conditions, to better understand trends and risk for severe disease. COVID-NET currently comprises 98 counties in 13 states. In addition, three large databases of electronic health care records (BD Insights Research Database [BD], the National Patient-Centered Clinical Research Network [PCORnet], and Premier Healthcare Database’s Special COVID-19 Release [Premier])*** will support monitoring of COVID-19 severity among hospitalized patients (i.e., percentages of patients in intensive care units [ICUs], those receiving invasive mechanical ventilation, and deaths).

Monitoring vaccination coverage, safety,^†††^ and VE are ongoing priorities because COVID-19–associated hospitalizations and deaths can be prevented through receipt of updated COVID-19 vaccines (*1*–*3*). Data use agreements established at the start of the pandemic with states, territories, and selected cities to facilitate receipt of comprehensive vaccine administration data from immunization information systems will terminate at the end of the public health emergency declaration. However, most jurisdictions have signed data use agreement extensions and will continue to submit COVID-19 vaccination data. Although future data might not be as complete because reporting requirements vary by state, the National Immunization Survey Child and Adult COVID Modules will continue to provide data on COVID-19 vaccination coverage and intent at the national and state levels via COVID Data Tracker and COVIDVaxView.^§§§^ VE platforms will continue to provide robust assessment of the real-world performance of vaccines (e.g., Investigating Respiratory Viruses in the Acutely Ill [IVY] and VISION Networks) (*1*,*2*). However, strategies for evaluating VE will need to incorporate alternative sources of vaccination data (e.g., patient/provider interviews or claims data) if immunization information systems are not sufficiently complete.

To continue facilitating access to national COVID-19 surveillance data, a first-phase, redesigned COVID Data Tracker website will launch on May 11, 2023. These data will continue providing an evidence base of information to guide prioritization of public health action. Numerous surveillance data sources and corresponding metrics and geographic levels will be updated weekly on COVID Data Tracker, with visualizations of trends and maps (Table 2). County-level hospitalization data will continue to include metrics on COVID-19–associated admissions and inpatient and ICU bed occupancy. Metrics for COVID-19–associated deaths (state-level), ED visits for COVID-19 (state-level), and percentage of positive SARS-CoV-2 test results (HHS region-level) will also be displayed. Metric levels will be anchored to levels of hospital admission rates used in the CCLs (*8*). The COVID Data Tracker will also continue to display SARS-CoV-2 variant proportion estimates and wastewater and traveler-based genomic surveillance data, as well as vaccination data and health care data on disease severity. In addition, availability of priority data will continue after May 11, 2023, for health equity, pediatric and special populations (e.g., vaccination coverage among persons who are pregnant and those with disabilities), health care settings (e.g., nursing home residents), and seroprevalence. SARS-CoV-2 infections remain nationally notifiable, and line-level COVID-19 case surveillance data will continue to be available, including public use data at https://data.cdc.gov.

## Data Collection That Will Be Discontinued After May 11, 2023

After the expiration of the public health emergency on May 11, 2023, authorizations to collect certain types of public health data expire (Table 1). The COVID Data Tracker includes a page for accessing archived data.^¶¶¶^ HHS can no longer require reporting of negative SARS-CoV-2 testing results via CELR reporting. This change removes the ability to monitor the national percentage of positive SARS-CoV-2 test results using the CELR data source. CELR data served as a useful early indicator of SARS-COV-2 transmission during the pandemic. However, since a peak of approximately 17.4 million NAATs performed weekly in January 2022, coinciding with the SARS-CoV-2 Omicron variant surge, the reported weekly volume of NAATs performed declined to less than 1 million by April 26, 2023. This decline is related in part to increased use of antigen tests as well as at-home testing.**** The CELR data have become more variable in quality or altogether unavailable in many jurisdictions over time. CDC’s COVID-19 Community Transmission Levels, which were derived, in part, from CELR data, also will be discontinued.

National reporting of aggregate weekly counts of COVID-19 cases and associated deaths, which CDC compiles using automated data extraction from jurisdictional websites and dashboards and direct submissions, will also be discontinued with the expiration of the public health emergency. This transition is consistent with many state and local health authorities’ decisions to discontinue public reporting of these data. Aggregate counts of COVID-19 cases have been useful for monitoring changing trends in incidence but have become less representative of actual rates of SARS-CoV-2 infections or levels of transmission over time, related to decreased laboratory testing, increased home testing, changes in reporting practices, and asymptomatic infections. Early in the pandemic, aggregate reporting from health departments provided more up-to-date counts of total deaths than did NVSS, but the timeliness of NVSS is now comparable with that of the aggregate counts (*8*,*9*). As part of the shift from reporting of aggregate death count data to use of NVSS data, date of death will be used rather than report date.

CCLs are based on a composite metric that includes COVID-19 hospital admission rates, inpatient bed utilization among patients with COVID-19, and case rates derived from aggregate reporting of case counts by jurisdictions. Because aggregate weekly case counts will end, CCLs also will end on May 11, 2023. Hospital admissions levels from NHSN closely align with CCLs (*8*) and will replace the CCL metric. Monthly reporting of case, hospitalization, and mortality rates by vaccination status will end with the expiration of the public health emergency.

## Discussion

Beginning in 2020, the historic response to the COVID-19 pandemic necessitated rapid improvements in processing, reporting, and visualizing of timely and granular public health surveillance data on an unprecedented scale. In 2023, as part of the transition of COVID-19 from emergency to routine public health program activities, CDC has established the Coronavirus and Other Respiratory Viruses Division,^††††^ which is committed to working with state, tribal, local, territorial, federal, and other partners on the prevention of COVID-19 within a sustainable and integrated surveillance strategy that monitors other circulating respiratory viruses and prevention measures, including vaccination, to provide timely and comprehensive situational awareness. In the past year, CDC has developed several public dashboards displaying data on hospitalizations or ED visits for diagnosed or laboratory-confirmed COVID-19, influenza, and respiratory syncytial virus.^§§§§^

Monitoring the impact of COVID-19 and the effectiveness of prevention and control strategies continues to be a public health priority during the transition from the emergency phase of the COVID-19 response to routine public health practice. Approximately 1,000 COVID-19–associated weekly deaths were reported in early April 2023; COVID-19–associated deaths are largely preventable through receipt of updated COVID-19 vaccine and timely administration of therapeutics^¶¶¶¶^ (*1*–4).

**Summary**

**What is already known about this topic?**

Authorizations to collect certain public health data expire at the end of the U.S. public health emergency declaration on May 11, 2023.

**What is added by this report?**

Changes to the national COVID-19 monitoring strategy and COVID Data Tracker capitalize on marked improvements in multiple surveillance systems. Weekly COVID-19 hospital admission levels and the percentage of all COVID-19–associated deaths will be primary surveillance indicators. Emergency department visits and percentage of positive SARS-CoV-2 laboratory test results will help detect early changes in trends. Genomic surveillance will continue to help identify and monitor SARS-CoV-2 variants.

**What are the implications for public health practice?**

COVID-19 is an ongoing public health problem that will be monitored with sustainable data sources to guide prevention efforts.

## Footnotes

*
https://aspr.hhs.gov/legal/PHE/

†
https://covid.cdc.gov/covid-data-tracker

§ https://www.cdc.gov/nchs/products/databriefs/db427.htm; https://www.cdc.gov/nchs/products/databriefs/db456.htm

¶ County-level hospital data, including new hospital admissions levels, are derived using calculations performed at the Health Service Area (HSA) level. An HSA is defined by CDC’s National Center for Health Statistics as a geographic area containing at least one county that is self-contained with respect to the population’s provision of routine hospital care. Every county in the United States is assigned to an HSA, and each HSA must contain at least one hospital. Data presented represent admissions and bed use among hospitals within the selected HSA.

**
https://www.cdc.gov/flu/weekly/index.htm#NCHSMortality

†† https://www.aphl.org/aboutAPHL/publications/Documents/ID-Influenza-Right-Size-Roadmap-Edition2.pdf; https://www.aphl.org/programs/preparedness/Crisis-Management/COVID-19-Response/Pages/Sequence-Based-Surveillance-Submission.aspx

§§ State, tribal, local, and territorial health departments participating in the NWSS submit testing data to CDC. CDC standardizes, interprets, and presents these data. How often sites collect wastewater samples and how frequently data are reported to CDC varies by health department. NWSS data for SARS-CoV-2 trends are updated daily, and data for SARS-CoV-2 variants are updated weekly (https://www.cdc.gov/nwss/index.html). The Traveler-Based Genomic Surveillance Program tracks SARS-CoV-2 variants by collecting samples from international air travelers arriving from more than 30 countries at seven major U.S. airports. These samples are then sent to a laboratory network for PCR testing; all positive samples undergo genomic sequencing. https://wwwnc.cdc.gov/travel/page/travel-genomic-surveillance

¶¶
https://www.cdc.gov/coronavirus/2019-ncov/covid-data/covid-net/purpose-methods.html

*** Data on disease severity among hospitalized COVID-19 patients are obtained from three large health care data sources that contain information from subsets of U.S. hospitals: BD, PCORnet, and Premier. Although none of these sources is national in scope, viewing trends across these three data sources adds to the overall understanding of COVID-19 disease severity. https://covid.cdc.gov/covid-data-tracker/index.html#hospitalizations-severity

††† https://www.cdc.gov/vaccinesafety/index.html

§§§ https://covid.cdc.gov/covid-data-tracker/#vaccine-confidence; https://www.cdc.gov/vaccines/imz-managers/coverage/covidvaxview/interactive.html

¶¶¶
https://covid.cdc.gov/covid-data-tracker/#archived

****
https://data.cdc.gov/Public-Health-Surveillance/U-S-COVID-19-Self-Test-Data/275g-9x8h

††††
https://www.federalregister.gov/documents/2023/02/13/2023-02930/establishment-of-the-coronavirus-and-other-respiratory-viruses-division

§§§§ https://www.cdc.gov/ncird/surveillance/respiratory-illnesses/index.html; https://www.cdc.gov/surveillance/resp-net/dashboard.html

¶¶¶¶
https://www.cdc.gov/coronavirus/2019-ncov/prevent-getting-sick/prevention.html
